# A simply calculated nutritional index provides clinical implications in patients undergoing transcatheter aortic valve replacement

**DOI:** 10.1007/s00392-023-02220-5

**Published:** 2023-05-13

**Authors:** Mitsumasa Sudo, Jasmin Shamekhi, Adem Aksoy, Baravan Al-Kassou, Tetsu Tanaka, Miriam Silaschi, Marcel Weber, Georg Nickenig, Sebastian Zimmer

**Affiliations:** 1 Heart Center Bonn, Department of Internal Medicine II, University Hospital Bonn, Venusberg-Campus 1, 53127 Bonn, Germany; 2 Division of Cardiology, Department of Medicine, Nihon University School of Medicine, Tokyo, Japan; 3 Heart Center Bonn, Department of Cardiac Surgery, University Hospital Bonn, Bonn, Germany

**Keywords:** Malnutrition, Transcatheter aortic valve replacement, Aortic stenosis, TCBI

## Abstract

**Background:**

Malnutrition is associated with adverse outcomes in patients with aortic stenosis. The Triglycerides × Total Cholesterol × Body Weight Index (TCBI) is a simple scoring model to evaluate the status of nutrition. However, the prognostic relevance of this index in patients undergoing transcatheter aortic valve replacement (TAVR) is unknown. This study aimed to evaluate the association of the TCBI with clinical outcomes in patients undergoing TAVR.

**Methods:**

A total of 1377 patients undergoing TAVR were evaluated in this study. The TCBI was calculated by the formula; triglyceride (mg/dL) × total cholesterol (mg/dL) × body weight (kg)/1000. The primary outcome was all-cause mortality within 3 years.

**Results:**

Patients with a low TCBI, based on a cut-off value of 985.3, were more likely to have elevated right atrial pressure (*p* = 0.04), elevated right ventricular pressure (*p* < 0.01), right ventricular systolic dysfunction (*p* < 0.01), tricuspid regurgitation ≥ moderate (*p* < 0.01). Patients with a low TCBI had a higher cumulative 3-year all-cause (42.3% vs. 31.6%, *p* < 0.01; adjusted HR 1.36, 95% CI 1.05–1.77, *p* = 0.02) and non-cardiovascular mortality (15.5% vs. 9.1%, *p* < 0.01; adjusted HR 1.95, 95% CI 1.22–3.13, *p* < 0.01) compared to those with a high TCBI. Adding a low TCBI to EuroSCORE II improved the predictive value for 3-year all-cause mortality (net reclassification improvement, 0.179, *p* < 0.01; integrated discrimination improvement, 0.005, *p* = 0.01).

**Conclusion:**

Patients with a low TCBI were more likely to have right-sided heart overload and exhibited an increased risk of 3-year mortality. The TCBI may provide additional information for risk stratification in patients undergoing TAVR.

**Graphical abstract:**

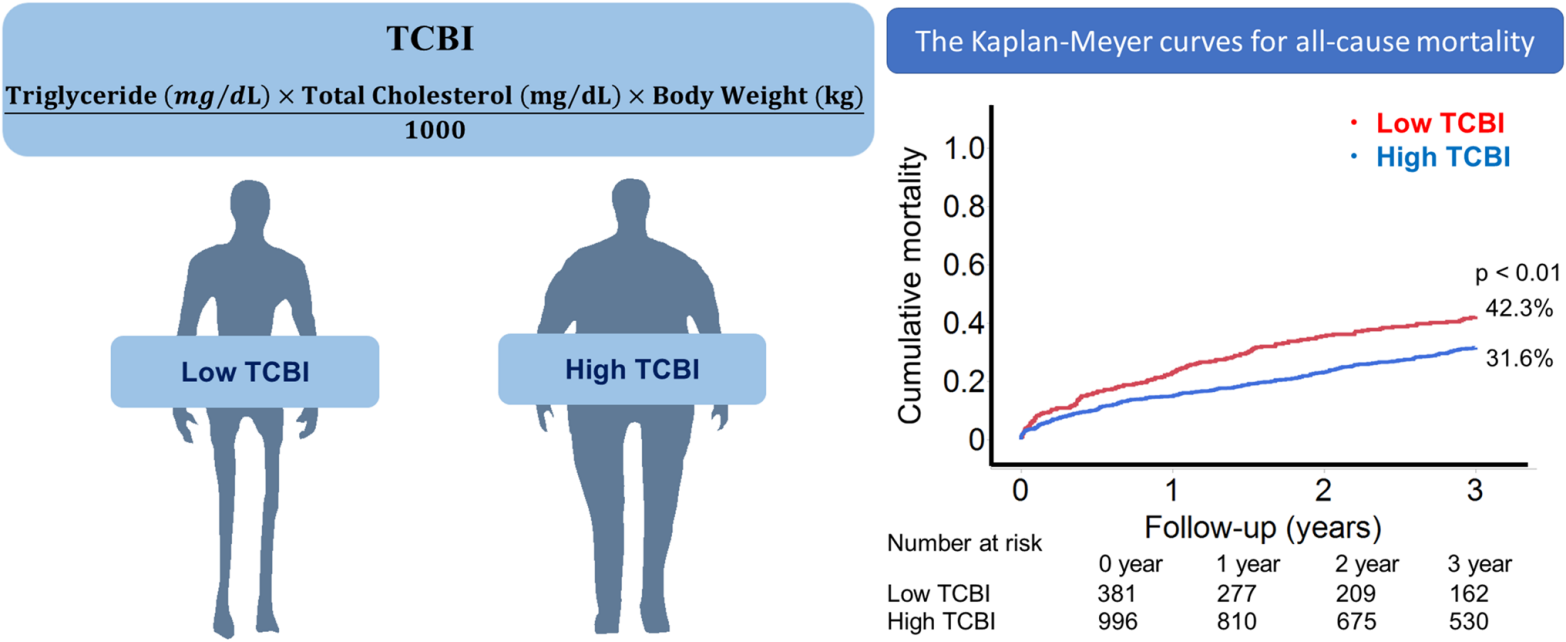

**Supplementary Information:**

The online version contains supplementary material available at 10.1007/s00392-023-02220-5.

## Introduction

Transcatheter aortic valve replacement (TAVR) is an established therapeutic option for patients with severe aortic stenosis and high surgical risk as an alternative to open heart surgery [1]. The evolution of device technology, interventional strategy, and patient care has greatly improved patient outcomes after TAVR [2]. Nevertheless, various comorbidities in patients with severe aortic stenosis undergoing TAVR impact prognosis. Of these, malnutrition is considered to be associated with frailty and is a risk factor not captured by traditional risk scores [3]. Therefore, consideration of the nutrition status before TAVR could play an important role in risk stratification and clinical management. To date, various nutrition scores have been developed, and malnutrition has been reported to be associated with prognosis in patients undergoing TAVR [4–6]. However, some of these formulas are complex and difficult to work in a clinical setting.

Recently, the Triglyceride × Total Cholesterol × Body Weight Index (TCBI) has been proposed as a simple to calculate nutrition score. This index was validated in patients with coronary artery disease and heart failure and was associated with an increased risk of worse outcomes [7–11]. Nonetheless, the relevance of the TCBI and its clinical implications in patients undergoing TAVR are unclear and need to be clarified. Thus, this study aimed to evaluate the association of the TCBI with clinical outcomes in patients undergoing TAVR.

## Methods

### Study population

The present study was conducted as a retrospective observational analysis of data from the TAVR registry Bonn, a single-center, observational, prospective cohort study. We reviewed the medical records of patients with symptomatic severe aortic stenosis who underwent TAVR at the Heart Center Bonn, University Hospital Bonn, between November 2008 and June 2019. The decision to perform TAVR was determined by the interdisciplinary heart team. Patients with missing data required to calculate the TCBI were excluded. This study was conducted according to the Declaration of Helsinki and with the approval of the institutional review board (No. 077/14). All patients provided written informed consent to the procedure and data acquisition.

### Assessment of the nutrition score

We routinely performed a blood examination at the time of admission before the TAVR procedure. The TCBI was retrospectively calculated based on the following formula [7]:

$$\mathrm{The TCBI}=\frac{\mathrm{Triglyceride }\left(\mathrm{mg}/\mathrm{dL}\right)\times \mathrm{ Total cholesterol }\left(\mathrm{mg}/\mathrm{dL}\right)\times \mathrm{Body weight }(\mathrm{kg}) }{1000}.$$

### Echocardiographic assessment

Echocardiographic assessments were performed before the TAVR procedure by two independently experienced physicians blinded to the results. All parameters were assessed in accordance with the current guidelines of the American Society of Echocardiography and the European Society of Echocardiography [12, 13]. Right ventricular systolic dysfunction was defined as a tricuspid annular plane systolic excursion < 1.7 cm. Elevated right ventricular pressure was defined as a tricuspid regurgitation pressure gradient ≥ 36 mmHg, and elevated right atrial pressure was defined as an inferior vena cava diameter ≥ 21 mm. Signs of right-sided heart overload were defined as right ventricular systolic dysfunction, elevated right ventricular pressure, elevated right atrial pressure, and moderate or worse tricuspid regurgitation [14, 15].

### Clinical outcomes

All clinical events were obtained retrospectively by examining medical records or telephone interviews. The primary outcome was defined as all-cause mortality within 3 years following TAVR. Secondary outcomes were cardiovascular mortality and non-cardiovascular mortality within 3 years.

### Statistical analysis

Categorical variables are presented as numbers with percentages. Continuous variables are presented as an average with standard deviation or median with an interquartile range (IQR). To assess a correlation between TCBI and Geriatric Nutritional Risk Index (GNRI), which was one of the conventional nutrition scores, the Spearman rank correlation coefficient was conducted. The GNRI was calculated according to the following formula: GNRI = 14.9 × serum albumin (g/dL) + 41.7 × body mass index/22 [16]. The receiver operating characteristic analysis was performed to determine the optimal cut-off value of the TCBI to predict all-cause mortality within 3 years after TAVR. Based on the cut-off value, patients were stratified into two groups: low TCBI and high TCBI. Inter-group differences in categorical variables were analyzed by using the Chi-square test or Fisher’s exact test. Inter-group differences in continuous variables were analyzed by unpaired Student’s t test or Wilcoxon rank-sum test. A logistic regression analysis was conducted to elucidate the association between a low TCBI and baseline demographic patient characteristics. A multivariable analysis was conducted using covariates of *p* < 0.10 in the univariate analysis.

The Kaplan–Meier method was used to estimate the cumulative mortality rate. A log-rank test was applied to compare the outcomes between the groups. Univariate and multivariable Cox proportional hazard regression analyses were conducted to calculate hazard ratios (HRs) and 95% confidence intervals (95%CIs) for the clinical outcomes. The association was adjusted in the multivariable model that included predefined covariates based on previous clinical knowledge as follows: sex, age, EuroSCORE II, chronic obstructive pulmonary disease, diabetes mellitus, New York Heart Association classification, atrial fibrillation, coronary artery disease, history of myocardial infarction, estimated glomerular filtration rate, hemoglobin, N-terminal prohormone of brain natriuretic peptide, serum albumin, left ventricular ejection fraction, tricuspid annular plane systolic excursion, mitral regurgitation, tricuspid regurgitation, and tricuspid regurgitation pressure gradient, based on previous clinical knowledge. Moreover, to clarify the association between each component of the TCBI and the primary outcome, a Cox proportional hazard analysis was conducted. In the multivariable analysis, covariates included each component of the TCBI. We depicted the association between the TCBI and the primary outcome using a spline curve. Net reclassification improvement and integrated discrimination improvement were calculated to evaluate the incremental effect of adding the TCBI to the EuroSCORE II on prediction of 3-year mortality.

Furthermore, potential interactions between the TCBI and following subgroups on the primary outcome were examined: sex (male vs. female), age (≥ 75 years vs. < 75 years), body surface area (≥ 1.68 m^2^ vs. < 1.68 m^2^), diabetes mellitus (no vs. yes), chronic obstructive pulmonary disease (no vs. yes), the New York Heart Association classification (≥ IIIvs. < III), EuroSCORE II (> 8% vs. ≤ 8%), serum albumin (≥ 3.5 g/dL vs. < 3.5 g/dL), renal function (estimated glomerular filtration rate ≥ 60 mL/min/1.73m^2^ vs. < 60 mL/min/1.73m^2^), left-ventricular function (left ventricular ejection fraction > 40% vs. ≤ 40%), right-ventricular function (tricuspid annular plane systolic excursion ≥ 1.7 cm vs. < 1.7 cm), severity of mitral regurgitation (≥ moderate vs. < moderate), statin (no vs. yes), and clinical frail scale (≥ 5 vs. < 5).

To examine the robustness of our inference, we performed a sensitivity analysis in which patients were divided into 3 groups based on tertile of the TCBI. A two-tailed *p* < 0.05 was accepted as statistically significant. All statistical analyses were performed using JMP 14 version 14.3.0 (SAS Institute Inc, Cary, NC, USA) and R version 4.1.1 (R Foundation for Statistical Computing, Vienna, Austria).

## Results

### Study population

A total of 1377 patients were analyzed in the present study. Overall, 49.9% of the patients were male, and mean age was 80.9 ± 6.1 months (Table 1). The median TCBI was 1460.2 (IQR 927.4, 2325.6), and the distribution of the TCBI is illustrated in Online Fig. 1. The TCBI positively correlated with the GNRI (*R* = 0.386, *p* < 0.01; Online Fig. 2). The optimal cut-off value of the TCBI for predicting 3-year all-cause mortality was 985.3 (Area under the curve, 0.55 [95%CI 0.52–0.59]; *p* < 0.01; Online Fig. 3). Based on this cut-off value, 381 patients (27.7%) were stratified into the low TCBI group, while 996 (72.3%) were stratified into the high TCBI group. Patients with a low TCBI were older (81.6 ± 6.2 years vs. 80.6 ± 6.0 years, *p* < 0.01), more likely to be male (54.6% vs. 48.1%, *p* = 0.03), and had more frequently diabetes mellitus (24.7% vs. 32.1, *p* < 0.01), atrial fibrillation (49.9% vs. 41.6%, *p* < 0.01), and statin use (74.9% vs. 65.2%, *p* < 0.01), compared to those with a high TCBI. The prevalence of clinical frail scale ≥ 5 was similar between two groups (Table 1). In multivariable logistic regression analysis, age (OR 1.03, 95%CI 1.00–1.05 [per 1 year increase], *p* = 0.02), atrial fibrillation (OR 1.31, 95%CI 1.01–1.70, *p* = 0.04), EuroSCORE II (OR 1.03, 95%CI 1.01–1.06 [per 1% increase], *p* = 0.01), and statin (OR 1.63, 95%CI 1.23–2.18, *p* < 0.01) were independently associated with a low TCBI. Moreover, diabetes mellitus was associated with reducing risk of a low TCBI in multivariable analysis (OR 0.65, 95%CI 0.48–0.87, *p* < 0.01) (Online Table 1).

**Table 1:** Patient characteristics

	Overall *n* = 1377	Low TCBI *n* = 381	High TCBI *n* = 996	*p* value
Baseline demographics				
Age, years	80.9 ± 6.1	81.6 ± 6.2	80.6 ± 6.0	< 0.01
Male, *n* (%)	687 (49.9)	208 (54.6)	479 (48.1)	0.03
Height, cm	167.6 ± 9.3	167.1 ± 9.3	167.8 ± 9.3	0.22
Body weight, kg	74 (64, 85)	70 (60, 78)	76 (66, 87)	< 0.01
Body surface area, mm^2^	1.85 (1.69, 1.97)	1.78 (1.62, 1.91)	1.87 (1.71, 1.87)	< 0.01
Body mass index, kg/m^2^	26.1 (23.4, 29.4)	24.2 (22.1, 26.5)	27.0 (24.1, 30.4)	< 0.01
Hypertension, *n* (%)	1185 (86.1)	319 (83.7)	866 (87.0)	0.12
Diabetes mellitus, *n* (%)	414 (30.1)	94 (24.7)	320 (32.1)	< 0.01
History of myocardial infarction, *n* (%)	172 (12.5)	54 (14.2)	118 (11.9)	0.24
Prior percutaneous coronary intervention, n (%)	508 (36.9)	150 (39.4)	358 (35.9)	0.24
Prior coronary artery bypass graft, *n* (%)	196 (14.2)	64 (16.8)	132 (13.3)	0.09
History of stroke, *n* (%)	164 (11.9)	47 (12.3)	117 (11.8)	0.76
COPD, *n* (%)	269 (19.5)	70 (18.4)	199 (20.0)	0.50
Hemodialysis, *n* (%)	42 (3.1)	11 (2.9)	31 (3.1)	0.83
Atrial fibrillation, *n* (%)	604 (43.9)	190 (49.9)	414 (41.6)	< 0.01
NYHA III or IV, *n* (%)	1257 (91.3)	356 (93.4)	901 (90.5)	0.08
EuroSCORE II (%)	4.48 (2.78, 7.66)	4.81 (2.85, 8.82)	4.32 (2.75, 7.27)	0.01
Clinical frail scale ≥ 5, *n* (%)	83 (30.3)	34 (41.0)	69 (36.1)	0.45
TCBI	1460.2 (927.4, 2325.6)	736.4 (568.4, 863.6)	1862.7 (1361.5, 2720.8)	< 0.01
Laboratory data				
Hemoglobin, mg/dL	11.6 ± 1.8	11.2 ± 1.8	11.8 ± 1.8	< 0.01
Albumin, mg/dL	3.9 (3.5, 4.2)	3.8 (3.4, 4.1)	4.0 (3.6, 4.2)	< 0.01
eGFR, mL/min/1.73m^2^	56.1 (41.8, 72.2)	56.8 (45.1, 76.4)	55.9 (41.1, 70.7)	0.02
Total cholesterol, mg/dL	166 (136, 201)	131 (112, 157)	181 (152, 213)	< 0.01
Triglyceride, mg/dL	120 (89, 165)	78 (66, 93)	141 (110, 188)	< 0.01
NT pro-BNP, pg/mL	2349 (912, 5821)	2971 (1191, 7905)	2105 (824, 4959)	< 0.01
Medial treatment				
Statin, *n* (%)	852 (67.9)	263 (74.9)	589 (65.2)	< 0.01
Echocardiographic parameters				
LV ejection fraction, %	55.0 ± 13.0	53.3 ± 14.4	55.6 ± 12.3	0.07
Mean aortic pressure gradient, mmHg	40.4 ± 15.2	40.1 ± 15.6	40.5 ± 15.0	0.69
Peak aortic pressure gradient, mmHg	70.4 ± 24.1	70.4 ± 25.2	70.5 ± 23.7	0.93
Aortic valve area, cm^2^	0.72 ± 0.16	0.70 ± 0.17	0.73 ± 0.16	0.01
Mitral regurgitation ≥ moderate, *n* (%)	640 (46.6)	209 (55.3)	431 (43.4)	< 0.01
Tricuspid regurgitation ≥ moderate, *n* (%)	324 (24.8)	121 (32.9)	203 (21.7)	< 0.01
TRPG, mmHg	33.1 ± 16.5	36.8 ± 16.7	31.7 ± 16.2	< 0.01
TAPSE, cm	2.0 (1.6, 2.4)	2.0 (1.5, 2.4)	2.1 (1.7, 2.4)	< 0.01
IVC, mm	14.1 ± 6.0	15.5 ± 6.3	13.5 ± 5.7	< 0.01

Categorical variables are presented as absolute numbers and percentages. Continuous variables are presented as the mean ± standard deviation or as the median and interquartile range

*COPD* chronic obstructive pulmonary disease; *eGFR* estimated glomerular filtration rate; *LV* left ventricular; *NYHA* New York Heart Association; *NT-pro BNP* N-terminal prohormone of brain natriuretic peptide; *TAPSE* tricuspid annular plane systolic excursion; *TCBI* Triglyceride × Total Cholesterol × Body Weight Index; *TRPG* tricuspid regurgitation pressure gradient

**Fig.1:**
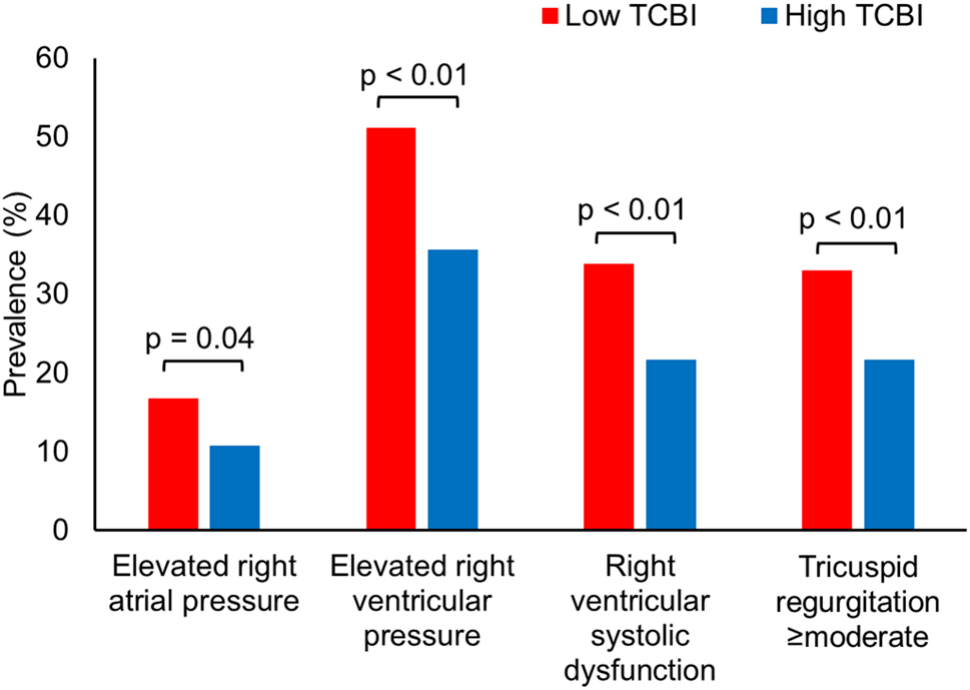
Association of TCBI with signs of right-sided heart overload. Patients with a low TCBI presented more frequently with elevated right atrial pressure (16.7% vs. 10.7, *p* = 0.04), elevated right ventricular pressure (51.1% vs. 35.6%, *p* < 0.01), right ventricular systolic dysfunction (33.8% vs. 21.6%, *p* < 0.01), and tricuspid regurgitation ≥ moderate (32.9% vs. 21.7%, *p* < 0.01). *TCBI* Triglyceride × Total Cholesterol × Body Weight Index

Regarding echocardiographic findings, patients with a low TCBI more frequently had signs of right-sided heart overload (elevated right atrial pressure [16.7% vs. 10.7%, *p* = 0.04], elevated right ventricular pressure [51.1% vs. 35.6%, *p* < 0.01], right ventricular systolic dysfunction [33.8% vs. 21.6%, *p* < 0.01], and tricuspid regurgitation ≥ moderate [32.9% vs. 21.7%, *p* < 0.01]), as compared to those with a high TCBI (Fig. 1).

### Clinical outcomes according to the TCBI

**Fig. 2:**
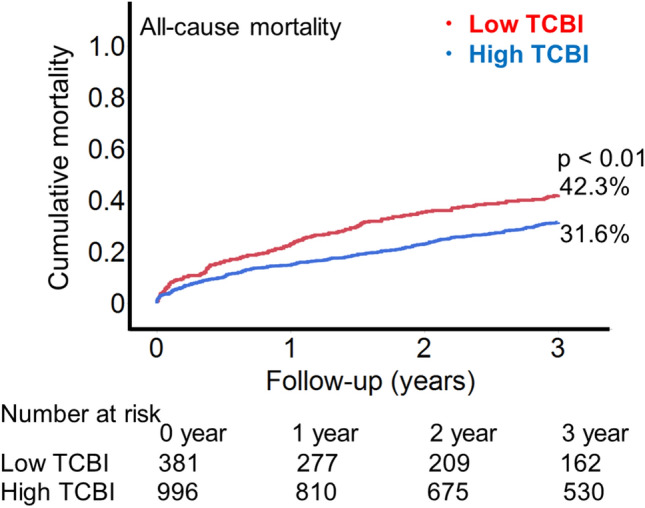
The Kaplan–Meyer curves for all-cause mortality. The Kaplan–Meyer curves showed that patients with a low TCBI had higher estimated 3-year all-cause mortality compared to those with a high TCBI (42.3% vs. 31.6%, log-rank *p* < 0.01). *TCBI* Triglyceride × Total Cholesterol × Body Weight Index

**Fig. 3:**
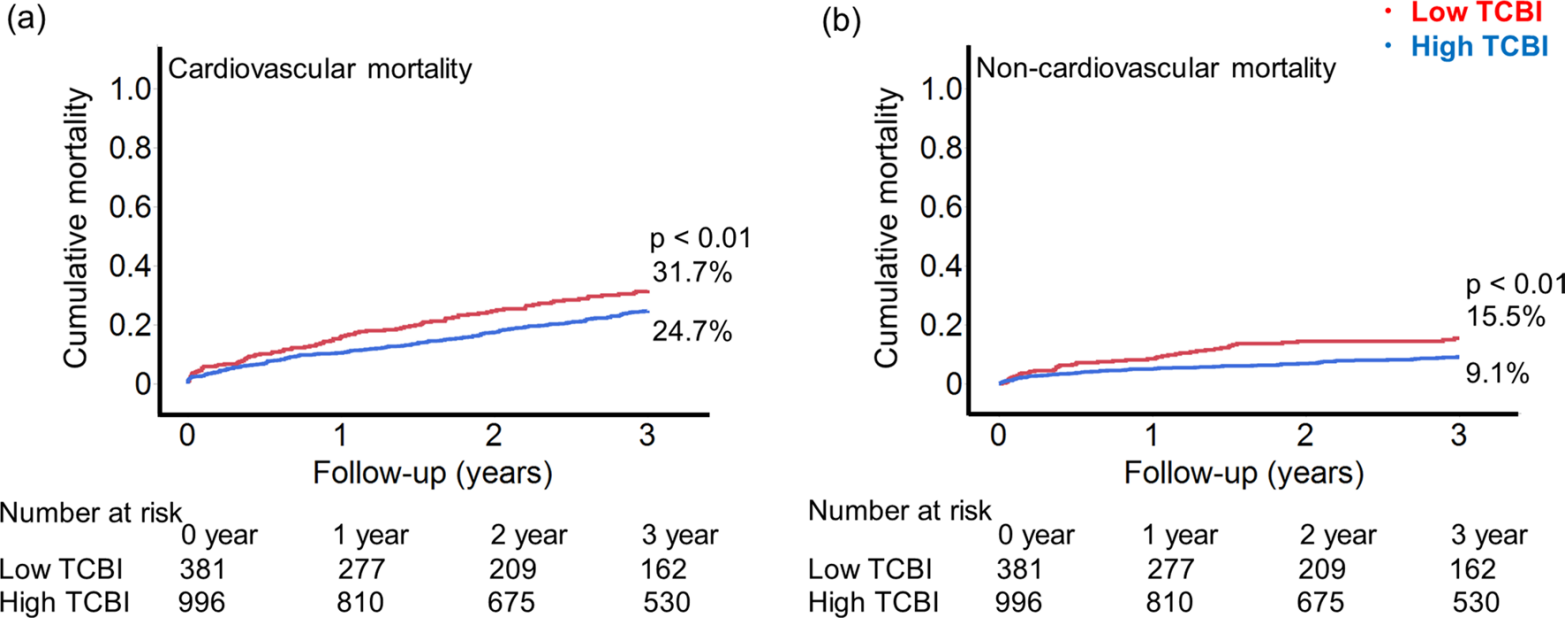
The Kaplan–Meyer curves for cardiovascular and non-cardiovascular mortality. At 3-year follow-up, patients with a low TCBI had higher rates of cardiovascular mortality (42.3% vs. 31.6%, log-rank *p* < 0.01) (**a**) and non-cardiovascular mortality (31.7% vs. 24.7%, log-rank *p* < 0.01) (**b**), as compared to patients with a high TCBI. *TCBI*, Triglyceride × Total Cholesterol × Body Weight Index

The median follow-up duration was 36.4 months (IQR 15.6, 55.6). The rate of cumulative mortality within 3 years was higher in patients with a low TCBI than in those with a high TCBI (42.3% vs. 31.6%, *p* < 0.01; Fig. 2). Similar associations were also observed within the first year (22.9% vs. 14.9%, *p* < 0.01) and the first two years (35.5% vs. 23.1%, *p* < 0.01). Furthermore, patients with a low TCBI showed higher rates of cardiovascular mortality (31.7% vs. 24.7%, *p* < 0.01) and non-cardiovascular mortality (15.5% vs. 9.1%, *p* < 0.01) within 3 years compared to those with a high TCBI (Fig. 3).

**Fig. 4:**
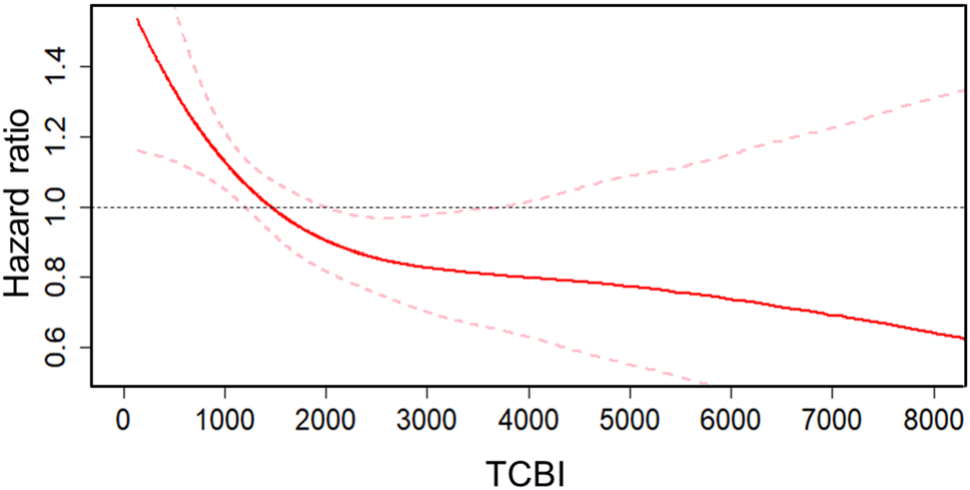
Spline curve with Cox hazard regression between the TCBI levels with 3-year all-cause mortality. The non-linear relationship between TCBI levels and the all-cause mortality demonstrated a consistently increasing hazard of the primary outcome with a lowering of the lower TCBI levels. Black dashed horizontal lines represent the hazard ratio of 1.0. Red lines indicate the estimated hazard ratio, and the pink dashed lines represent a 95% confidence interval. *TCBI* Triglyceride × Total Cholesterol × Body Weight Index

**Fig. 5:**
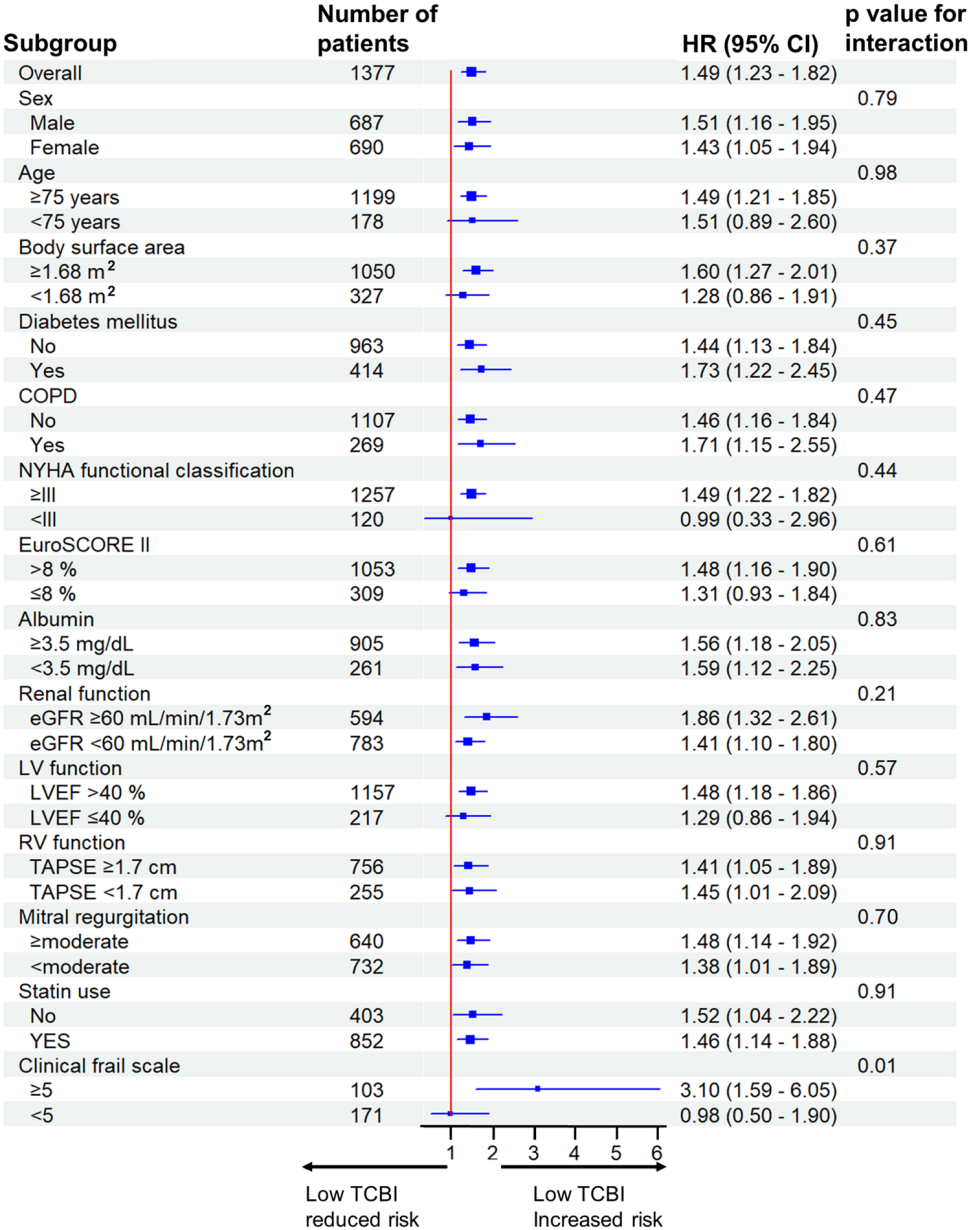
Subgroup analysis of the primary outcome in patients with a low TCBI. A Forest plot illustrates hazard ratios for 3-year primary outcome after TVAR in patients with a low TCBI. In each subgroup, hazard ratio and 95% confidence intervals are presented. *CI* confidence interval; *COPD* chronic obstructive pulmonary disease; *HR* hazard ratio; *LV* left ventricular; *LVEF* left ventricular ejection fraction; *NYHA* New York Heart Association; *RV* right ventricular; *TCBI* Triglyceride × Total Cholesterol × Body Weight Index; *TAPSE* tricuspid annular plane systolic excursion; *TAVR* transcatheter aortic valve replacement

In a univariate Cox regression hazard model, a low TCBI was associated with a higher risk of all-cause mortality (HR 1.49, 95%CI 1.23–1.82, *p* < 0.01) (Table 2), which was consistent in a multivariable model (adjusted HR 1.36, 95%CI 1.05–1.77, *p* = 0.02). Similarly, the TCBI as a continuous value was associated with all-cause mortality (unadjusted HR 0.75, 95%CI 0.65–0.86 [per 1 log TCBI increase], *p* < 0.01; adjusted HR 0.81, 95%CI 0.67–0.99 [per 1 log TCBI increase], *p* = 0.04). The association between the TCBI and all-cause mortality within 3 years was depicted using a spline curve (Fig. 4). The association between the TCBI and all-cause mortality was consistent across clinical subgroups, including body surface area and statin use, except for clinical frail scale ≥ 5 (Fig. 5).

**Table 2:** Association of a low TCBI with primary and secondary outcomes

	Univariate analysis		Multivariable analysis	
	HR (95% CI)	*p* value	HR (95% CI)	*p* value
Primary outcome				
All-cause mortality	1.49 (1.23–1.82)	< 0.01	1.36 (1.05–1.77)	0.02
Secondary outcomes				
Cardiovascular mortality	1.38 (1.09–1.75)	< 0.01	1.17 (0.85–1.61)	0.34
Non-cardiovascular mortality	1.81 (1.26–2.60)	< 0.01	1.95 (1.22–3.13)	< 0.01

The association was adjusted in the multivariable model that included predefined covariates as follows: sex, age, EuroSCORE II, chronic obstructive pulmonary disease, diabetes mellitus, New York Heart Association classification, atrial fibrillation, coronary artery disease, history of myocardial infarction, estimated glomerular filtration rate, hemoglobin, N-terminal prohormone of brain natriuretic peptide, serum albumin < 3.5 mg/dL, left ventricular ejection fraction, tricuspid annular plane systolic excursion, mitral regurgitation, tricuspid regurgitation, and tricuspid regurgitation pressure gradient

*CI* confidence interval; *HR* hazard ratio; *TCBI* Triglyceride × Total Cholesterol, × Body Weight Index

Also, a low TCBI was associated with the risk of non-cardiovascular mortality (adjusted HR 1.95, 95%CI 1.22 – 3.13, *p* < 0.01), while the association between the TCBI and cardiovascular mortality was not significant in the multivariable model. Regarding the association between TCBI components and primary outcome, total cholesterol was independently associated with all-cause mortality in the multivariable analysis (Online Table 2).

Furthermore, adding a low TCBI to EuroSCORE II improved the prediction of the 3-year all-cause mortality (net reclassification improvement, 0.179, *p* < 0.01; integrated discrimination improvement, 0.005, *p* = 0.01).

### Sensitivity analysis

Patients were stratified into three groups based on tertiles of the TCBI: the first tertile (TCBI ≤ 1077.1), the second tertile (1077.1 < TCBI ≤ 1947.2), and the third tertile (TCBI > 1947.2). Kaplan–Meyer curves showed that the cumulative 3-year all-cause mortality rate was higher in the first tertile than in the combined second and third tertiles (39.0% vs. 32.3%, *p* = 0.01; Online Fig. 4).

## Discussion

The present study is the first to assess the clinical implications of the TCBI, a simple to calculate nutrition marker, in patients undergoing TAVR. The findings in this observational study are as follows:

1. A low TCBI was associated with elevated right atrial pressure, elevated right ventricular pressure, right ventricular systolic dysfunction, and tricuspid regurgitation ≥ moderate.
2. The TCBI was independently associated with all-cause and non-cardiovascular mortality within 3 years after TAVR.
3. Adding the TCBI to the EuroSCORE II improved the prediction of the 3-year all-cause mortality.

Patients with severe aortic stenosis are often elderly, and comorbidities such as frailty and malnutrition were associated with a risk of adverse outcomes for open heart surgery [17]. Even in the established TAVR as an invasive therapeutic option, malnutrition is considered a risk that a conventional risk score cannot capture [3]. Assessing an adequate nutritional status is significant for risk stratification in patients undergoing TAVR. Although various nutrition scores have been proposed and were associated with worse outcomes [4–6], some of these scores are complex, and no consensus has yet been reached on a nutrition score in patients undergoing TAVR in clinical practice. Therefore, it is worthwhile that the TCBI, a simple to calculate nutrition marker, has shown an association with clinical outcomes in the present study.

Among composing factors of the numerous nutrition scores, lipid profiles often are used as lipid metabolism, and the body weight or body mass index is used to quantify muscle mass and fat mass. Taken together, these factors are associated with caloric depletion and preservation. Conventional nutrition assessments, such as the geriatric nutritional risk index and the controlling nutritional status score, were calculated using these parameters [6]. Thus, the TCBI has been developed to reflect the nutrition status of a patient by simply multiplying total glyceride, total cholesterol, and body weight. Previous studies reported that the dual X-ray absorptiometry scan, the gold standard assessment of nutritional status, correlated with the GNRI [18, 19]. In line with a previous study, our findings showed a positive correlation between TCBI and GNRI [10, 11]. Thus, the TCBI may indirectly reflect nutritional status.

In the present study, a low TCBI was associated with signs of right-sided heart overload. Mechanistically, persistently elevated left ventricular pressure in aortic stenosis patients can lead to increased pulmonary artery pressure and right-sided heart-filling pressure. Chronic right-sided heart overload is related to right ventricular dysfunction and tricuspid regurgitation [20, 21]. Patients with a low TCBI were more likely to be MR ≥ moderate and tended to lower left ventricular ejection fraction than those with a high TCBI, which might facilitate right-sided heart-filling pressure. Sze et al. reported that intestinal edema resulting from right-sided heart overload in chronic heart failure could interfere with nutrient absorption, promoting malnutrition [15]. Thus, it is conceivable that a low TCBI is related to right-sided heart overload.

In line with previous studies of coronary artery disease and acute decompensated heart failure, a low TCBI was associated with all-cause mortality in patients undergoing TAVR [7, 10]. Similarly, our finding is consistent with previous studies, which have been reported worse prognosis after TAVR in malnourish patients, as assessed by other nutrition scores [4–6]. A novel aspect of our study is that such a simply calculated nutrition score as TCBI showed an independent risk for all-cause mortality in multivariable, non-linear spline, subgroup, and sensitivity analyses. Furthermore, adding a low TCBI to the conventional surgical risk score improved the predictive value for 3-year all-cause mortality.

In the present study, non-cardiovascular mortality was associated with a low TCBI. It is well-established that nutritional status is related to immune function; and malnutrition decreases lymphocytes and impairs the immune system. [22, 23]. Our findings of increased non-cardiac mortality, which includes, infection and carcinoma, in patients with a low TCBI, are in line with previous studies [22, 24, 25]. However, a low TCBI was not associated with cardiac mortality in the multivariable Cox proportional hazard regression analysis. One possible explanation is that patients with a low TCBI more often suffered from mitral regurgitation ≥ moderate, and right-sided heart overload than patients with a high TCBI, leading to adverse cardiac events [26, 27]. As a result, the TCBI might not have remained independently associated with cardiac mortality in multivariable analysis.

Obesity and hyperlipidemia are known risk factors of coronary atherosclerosis and cardiac death [28, 29]. However, non-linear spline analysis showed that increased TCBI was not associated with all-cause mortality in the present study. One of the reasons might be that coronary artery disease is often treated prior to TAVR and most of these patients are treated with statins, which can stabilize atherosclerotic plaque and prevent ischemic events [30, 31]. Hence, an increased TCBI might not have been associated with all-cause mortality within 3 years following TAVR. This finding supports the importance of assessing malnutrition and overnutrition in patients undergoing TAVR.

Subgroup analyses consistently reveal a similar prognostic implication for the TCBI, except for clinical frail scale ≥ 5. Especially, a low TCBI has been associated with mortality, irrespective of statin use, in line with a previous study [7]. This finding suggests that the TCBI can help predict outcomes even in patients with a cardiac disease taking statins which decrease lipid levels. Therefore, the TCBI might provide additional information for risk stratification in patients undergoing TAVR.

In the present study, the area under the curve was relatively low value. One possible explanation might be that the follow-up needed to be completed. Hence, the Kaplan–Meyer methods, multivariable Cox hazard proportion analysis, spline curve, and subgroup analysis were performed to evaluate the clinical implication of a low TCBI. It was a worthwhile finding that a low TCBI remained independently associated with all-cause mortality in multivariable analysis.

Recent studies reported that patients with improved malnutrition after TAVR had better survival rates than patients with remained malnourished [32, 33]. Considering it, knowing a low TCBI before the procedure may allow for identifying patients for necessary nutritional intervention and improve outcomes. Since the follow-up TCBI was not measured in the present study, further study needs to clarify whether improvement in TCBI impacts mortality.

## Limitations

Several limitations should be acknowledged in the present study. First, since this study evaluated the relevance of the TCBI in a single-center cohort study, our findings might be subjected to selection bias. Second, the TCBI was not followed up after TAVR. Third, clinical frail scale was available in a few patients. Further study is needed to clarify the association between frail status and the TCBI and to validate our findings. Nevertheless, this is the first study to evaluate the association of the TCBI, a simple to calculate nutrition marker, with clinical outcomes after TAVR.

## Conclusion

A low TCBI was associated with signs of right-sided heart overload exhibited an increased risk of 3-year mortality. In addition, adding the TCBI to EuroSCORE II improved the predictive value for all-cause mortality. Our findings could provide additional information for the risk assessment of patients undergoing TAVR.

### Supplementary Information

Below is the link to the electronic supplementary material.

Supplementary file1 (DOCX 504 kb)
