# Supplementary material for: A simply calculated nutritional index provides clinical implications in patients undergoing transcatheter aortic valve replacement
Source: Clin Res Cardiol. 2023 May 13;113(1):58–67. doi: 10.1007/s00392-023-02220-5 (PMC10808226; doi:10.1007/s00392-023-02220-5)
Supplement: Supplementary file 1 — Supplementary file1 (DOCX 504 kb) [file 392_2023_2220_MOESM1_ESM.docx]

**Supplemental Information**

**A simply calculated nutritional index provides clinical implications in patients undergoing transcatheter aortic valve replacement**

Mitsumasa Sudo, MD ^1, 2^; Jasmin Shamekhi, MD ^1^; Adem Aksoy, MD ^1^; Baravan Al-Kassou, MD ^1^; Tetsu Tanaka, MD ^1^, Miriam Silaschi, MD ^3^; Marcel Weber, MD ^1^; Georg Nickenig, MD ^1^; Sebastian Zimmer, MD ^1^

1 Heart Center Bonn, Department of Internal Medicine II, University Hospital Bonn, Germany

2 Division of Cardiology, Department of Medicine, Nihon University School of Medicine, Tokyo, Japan

3 Heart Center Bonn, Department of Cardiac Surgery, University Hospital Bonn, Germany

**Address for correspondence:**

Mitsumasa Sudo, MD

Heart Center Bonn, Department of Medicine II, University Hospital Bonn

Venusberg-Campus 1, 53127 Bonn, Germany

Phone +49-228-287-16139, Fax +49-228-287-14983

E-mail address: m.s-sudo@fine.ocn.ne.jp

**Online Table 1. Association of baseline demographic patient characteristics with a low TCBI**

|  | Univariate analysis | | Multivariable analysis | |
| --- | --- | --- | --- | --- |
|  | OR (95% CI) | p value | OR (95% CI) | p value |
| Age (per 1 year increase) | 1.03 (1.01 – 1.05) | 0.04 | 1.03 (1.00 – 1.05) | 0.02 |
| Male | 1.30 (1.02 – 1.64) | 0.03 | 1.23 (0.95 – 1.61) | 0.12 |
| Diabetes mellitus | 0.69 (0.53 – 0.90) | <0.01 | 0.65 (0.48 – 0.87) | <0.01 |
| Atrial fibrillation | 1.40 (1.10 – 1.77) | <0.01 | 1.31 (1.01 – 1.70) | 0.04 |
| CABG | 1.32 (0.95 – 1.83) | 0.09 | 0.94 (0.61 – 1.45) | 0.78 |
| NYHA III or IV | 1.50 (0.95 – 2.37) | 0.08 | 1.35 (0.83 – 2.18) | 0.22 |
| EuroSCORE II (per 1% increase) | 1.04 (1.02 – 1.06) | <0.01 | 1.03 (1.01 – 1.06) | 0.01 |
| Statin | 1.60 (1.21 – 2.11) | <0.01 | 1.63 (1.23 – 2.18) | <0.01 |

A multivariable analysis was conducted using covariates of p<0.10 in the univariate analysis.

Abbreviations: CABG: coronary artery bypass graft; CI, confidence interval; NYHA, New York Heart Association; OR, odds ratio.

**Online Table 2. Association of all-cause mortality with components of the TCBI**

| Components of the TCBI | Univariable analysis | | Multivariable analysis | |
| --- | --- | --- | --- | --- |
|  | HR (95% CI) | p value | HR (95% CI) | p value |
| Triglyceride (per 1 mg/dL increase) | 0.999 (0.998 – 1.000) | 0.17 | 1.000 (0.999 – 1.001) | 0.56 |
| Total Cholesterol (per l mg/dL increase) | 0.994 (0.992 – 0.996) | <0.01 | 0.993 (0.991 – 0.996) | <0.01 |
| Body Weight (per 1mg/dL increase) | 0.998 (0.993 – 1.001) | 0.62 | 0.989 (0.989 – 1.001) | 0.13 |

Abbreviations: CI, confidence interval; HR, hazard ratio; TCBI, Triglyceride × Total Cholesterol × Body Weight Index

**Online Fig. 1. The distribution of the TCBI**

**
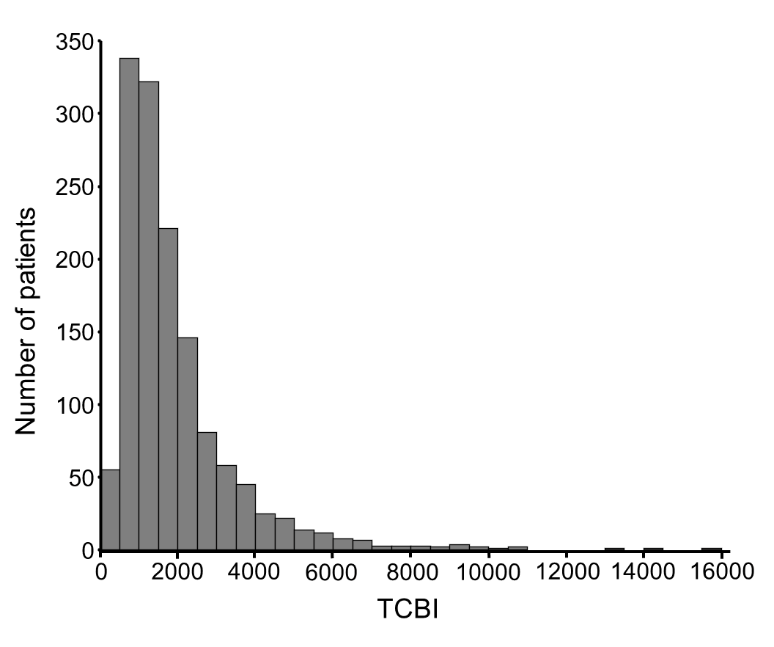
**

Abbreviations: TCBI, Triglyceride × Total Cholesterol × Body Weight Index.

**Online Fig. 2. Correlation between the TCBI and the GNRI.**

**
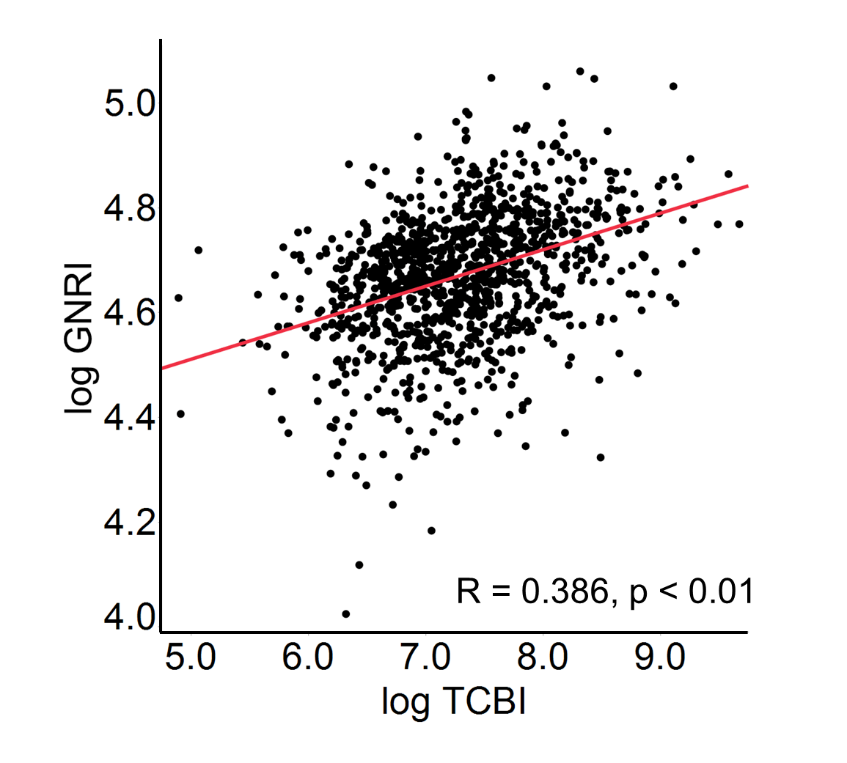
**

Abbreviations: GNRI, Geriatric Nutritional Risk Index; TCBI, Triglyceride × Total Cholesterol × Body Weight Index.

**Online Fig. 3. The receiver operating characteristic curve of the TCBI for predicting all-cause death within three years after TAVR**

**
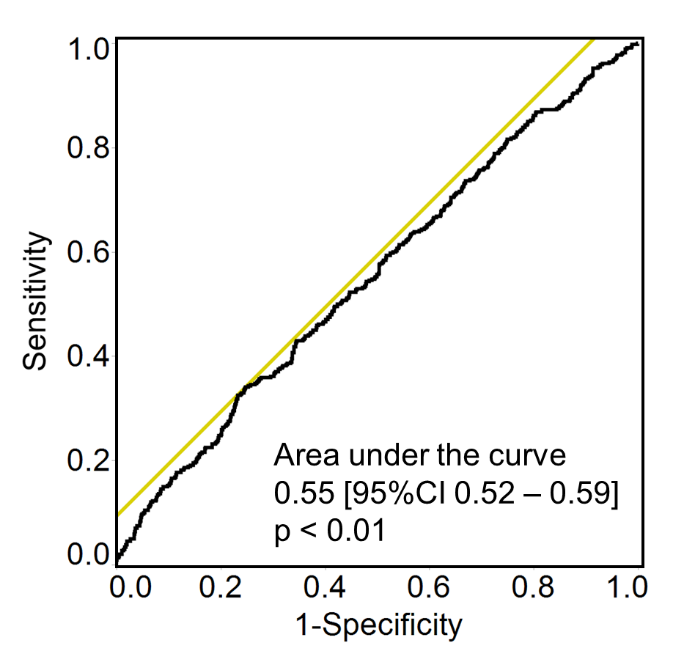
**

The area under the curve was 0.55 (95%CI 0.52 – 0.59, p < 0.01). The optimal cut-off value of the TCBI for predicting three-year all-cause death was 985.3.

Abbreviations: CI, confidence interval; TCBI, Triglyceride × Total Cholesterol × Body Weight Index; TAVR, transcatheter aortic valve replacement.

**Online Fig. 4. The Kaplan-Meyer curves for all-cause mortality based on tertiles of the TCBI**

**
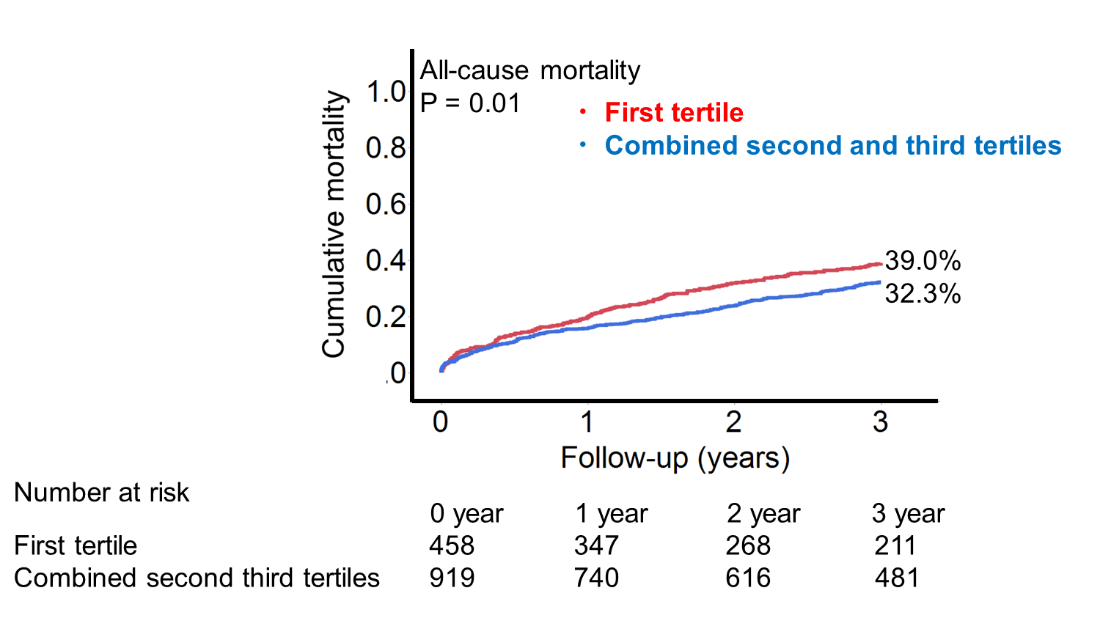
**

The Kaplan-Meyer curves showed that the cumulative three-year all-cause mortality rate was higher in the first tertile than in the combined second and third tertiles (39.0% vs. 32.3%, p = 0.01).

Abbreviations: TCBI, Triglyceride × Total Cholesterol × Body Weight Index
